# Combining nanoscale zero-valent iron and anaerobic dechlorinating bacteria to degrade chlorinated methanes and 1,2-dichloroethane

**DOI:** 10.1007/s11356-023-25376-z

**Published:** 2023-01-27

**Authors:** Dani Salom, David Fernández-Verdejo, Javier Moral-Vico, Xavier Font, Ernest Marco-Urrea

**Affiliations:** Departament d’Enginyeria Química, Biològica i Ambiental, Universitat Autònoma de Barcelona (UAB), 08193 Bellaterra, Barcelona, Spain

**Keywords:** Nanoscale zero-valent iron, Organohalide respiring bacteria, *Dehalobacterium*, *Dehalogenimonas*, Chlorinated organic pollutants

## Abstract

Nanoscale zero-valent iron (nZVI) has the potential to degrade a diversity of chlorinated compounds, and it is widely used for remediation of contaminated groundwaters. However, some frequently detected contaminants such as dichloromethane (DCM) and 1,2-dichloroethane (1,2-DCA) have shown nearly no reactivity with nZVI. Here, we tested the feasibility of combining anaerobic dechlorinating bacteria, *Dehalobacterium* and *Dehalogenimonas*, and nZVI as a treatment train to detoxify chlorinated methanes (i.e., chloroform-CF- and DCM), and 1,2-DCA. First, we showed that CF (500 μM) was fully degraded by 1 g/L nZVI to DCM as a major by-product, which was susceptible to fermentation by *Dehalobacterium* to innocuous products. Our results indicate that soluble compounds released by nZVI might cause an inhibitory impact on *Dehalobacterium* activity, avoiding DCM depletion. The DCM dechlorination activity was recovered when transferred to a fresh medium without nZVI. The increase in H_2_ production and pH was discarded as potential inhibitors. Similarly, a *Dehalogenimonas*-containing culture was unable to dichloroeliminate 1,2-DCA when exposed to 1 g/L nZVI, but dechlorinating activity was also recovered when transferred to nZVI-free media. The recovery of the dechlorinating activity of *Dehalobacterium* and *Dehalogenimonas* suggests that combination of nZVI and bioremediation techniques can be feasible under field conditions where dilution processes can alleviate the impact of the potential inhibitory soluble compounds.

**Supplementary Information:**

The online version contains supplementary material available at 10.1007/s11356-023-25376-z.

## Introduction

Nanoscale zero-valent iron (nZVI) is an emerging technology to remediate groundwaters polluted with chlorinated organic compounds, which are persistent and toxic pollutants that threaten the environment and health (Koenig et al. 2015; Wang et al. 2016; Zhang 2003; El-Shahawi et al. 2010). Reductive dehalogenation carried out by nZVI is a surface-mediated reaction in which electrons are transferred from nZVI to organochlorines and produce lesser chlorinated compounds and various oxides on the surface with the release of Cl^−^ and H_2_ (Wang and Zhang 1997; Zhang 2003).

The use of nZVI has been encouraged due to its greater specific surface area and higher degradation rates in comparison with conventional iron powder (Wang and Zhang 1997). In addition, nZVI allows in situ groundwater treatments by direct injection under pressure or by gravity into groundwater, which is preferred to ex situ treatments (i.e., pump and treat) because of the lower cost and energy consumption, reduced worker exposure, and limited site disruption (Elliott and Zhang 2001; Wang et al. 2016).

Despite the reactivity of nZVI toward a broad spectrum of chlorinated compounds, dichloromethane (DCM) and 1,2-dichloroethane (1,2-DCA) have shown nearly no reactivity with nZVI (Koenig et al. 2016; Song and Carraway 2006; Song and Carraway 2005; Tobiszewski and Namiesnik 2012). This presents a problem for the remediation of groundwater contaminated with complex chemical mixtures containing DCM and 1,2-DCA as primary pollutants. Interestingly, another abiotic source of DCM in groundwaters is the hydrogenolysis of chloroform (CF) by nZVI, which accumulates in the medium with no further degradation (Song and Carraway 2005). In this scenario, the approach of coupling nZVI treatments with functional anaerobic dechlorinating bacteria is promising to fully detoxify such chlorinated compounds (Chen et al. 2014; Liu et al. 2022; Kocur et al. 2015; Kocur et al. 2016; Wang et al. 2016), but the effect of nZVI on bacterial degraders has been poorly explored yet.

Due to the frequent anoxic conditions of contaminated groundwaters, organohalide-respiring bacteria (ORB) are commonly used for the detoxification of chlorinated compounds. At the heart of this process are membrane-bound reductive dehalogenases, which use chlorinated compounds as electron acceptors to generate cellular energy while using H_2_ as electron donor (Koenig et al. 2015; Richardson 2013; Wang et al. 2016). As for nZVI, enhanced biological reductive dechlorination has been proven to be an effective in situ treatment to remove chlorinated compounds in anoxic groundwaters with a relatively low cost compared to other physicochemical techniques (Chen et al. 2015; Blázquez-Pallí et al. 2019). To date, dichloroelimination of 1,2-DCA to ethene under anaerobic conditions has been demonstrated for several ORB, including *Dehalococcoides mccartyi*, *Dehalogenimonas*, *Desulfitobacterium*, *Sulfurospirillum*, and *Dehalobacter* (De Wildeman et al. 2003; Maymó-Gatell et al. 1999; Moe et al. 2009; Grostern and Edwards 2006; van der Zaan et al. 2009). On the other hand, anaerobic DCM degradation is a relatively novel pathway for which only three bacteria, all belonging to *Peptococcaceae* family, have been identified: “*Candidatus* Formimonas warabiya” (formerly referred as strain DCMF), *Dehalobacterium formicoaceticum*, and “*Candidatus* Dichloromethanomonas elyuquensis” (Mägli et al. 1998; Chen et al. 2017a; Kleindienst et al. 2019; Holland et al. 2021; Trueba-Santiso et al. 2020).

To adequately detoxify sites with complex mixtures containing CF and 1,2-DCA, a combined strategy including both nZVI and anaerobic dechlorinating bacteria can be of interest. For instance, CF has been reported to inhibit microbial reductive dechlorination of chloroethenes, chloroethanes, chlorophenols, and DCM (Bagley et al. 2000; Trueba-Santiso et al. 2020; Futagami et al. 2013), but nZVI might alleviate such inhibition by decreasing CF concentration yielding DCM. On the other hand, the use of nZVI can benefit ORB by creating the strong reducing conditions required for their growth and generating H_2_ as electron donor for organohalide respiration (Kocur et al. 2015; Kocur et al. 2016; Koenig et al. 2016; Xiu et al. 2010). At the same time, anaerobic dechlorinating bacteria can detoxify 1,2-DCA and DCM, which showed almost no reactivity with nZVI. However, the presence of nZVI can be detrimental to microbial reductive dechlorination. Reasons for this nZVI toxicity are not still clear. nZVI particles can interact with cell membranes causing disturbances in cell membrane integrity and interfere with respiration due to their reducing power, and extensive DNA and protein damage by the generation of reactive oxygen species (ROS) if nanoparticles enter into the cytoplasm (Koenig et al. 2015; Rangan et al. 2020; Saccà et al. 2014; Wang et al. 2016; Xie et al. 2017).

In this work, we investigated the impact of nZVI on two bacterial models capable of detoxifying DCM and 1,2-DCA. First, we intend to evaluate the feasibility of a treatment train to transform CF to DCM via nZVI and the subsequent fermentation of DCM to acetate via a *Dehalobacterium*-containing culture. For this, the effect of different concentrations of nZVI on the chemical reduction of CF and the inhibition of *Dehalobacterium* was studied. Second, 1,2-DCA has shown no reactivity with nZVI, but it can be used as electron acceptor by the ORB belonging to the *Dehalogenimonas* genus. Therefore, we studied the inhibitory effect of nZVI on a *Dehalogenimonas*-containing culture that transformed 1,2-DCA to ethene. This study provides valuable information to delineate remediation strategies that combine both anaerobic dechlorinating bacteria and nZVI at contaminated sites.

## Materials and methods

### nZVI synthesis and bacterial cultures

Particles of nZVI were synthesized as described elsewhere (Wang and Zhang 1997). Briefly, nZVI was synthesized using a 3.6 M sodium borohydride (NaBH_4_, ≥ 98%, Sigma-Aldrich) aqueous solution and a 0.9 M ferrous chloride (III) (FeCl_3_, ≥ 98%, Sigma-Aldrich) aqueous solution. Both solutions were prepared using ultrapure water (Milli-Q). Water used to prepare the FeCl_3_ solution was previously deoxygenated by bubbling with N_2_. Then, the NaBH_4_ solution was added dropwise to the FeCl_3_ solution while stirring at 200 rpm and bubbling with N_2_. Then, nZVI was washed three times using anaerobic ultrapure water, and it was finally stored in a serum bottle and sealed with Teflon-coated butyl rubber aluminum crimp caps until use to keep anaerobic conditions.

The cultures containing *Dehalogenimonas* and *Dehalobacterium *derived from the Besòs River (Barcelona, Spain) estuary sediments and slurry samples collected from a membrane bioreactor operating in a centralized industrial wastewater treatment plant, respectively. Both cultures were cultivated for more than 5 years in an anaerobic defined medium described elsewhere (Trueba-Santiso et al. 2017; Martín-González et al. 2015). Briefly, both media contained vitamins and trace elements (KH_2_PO_4_, NH_4_Cl, CaCl_2_, MgCl_2_·6H_2_O, KCl, CaCl_2_·2H_2_O, NTA, FeCl_2_·4H_2_O, ZnCl_2_, MnCl_2_·2H_2_O, CoCl_2_·6H_2_O, H_3_BO_3_, NiCl_2_·6H_2_O, and NaMoO_4_·H_2_O) and Na_2_S·9H_2_O and L-cysteine (0.2 mM each) as reducing agents. *Dehalobacterium* cultures also contained tungsten (22.8 μM) and selenium (24.2 μM). Yeast extract (200 mg/L) and acetate (5 mM) were added to the *Dehalobacterium* and *Dehalogenimonas* cultures, respectively. Microcosms containing *Dehalobacterium* were gassed with N_2_ (0.4 bar overpressure), and *Dehalogenimonas* cultures were gassed with N_2_/CO_2_ (4:1, v/v, 0.2 bar overpressure) and H_2_ (added to an overpressure of 0.4 bar) unless otherwise stated. Both media were buffered with bicarbonate solution (0.01 M). When indicated, either Tris-HCl (25 mM, pH 7.5) or HEPES (60 mM) was added as buffer solutions.

### Establishment of microcosms

Sixty-five mL of the anaerobic medium described above was added into 100-mL serum bottles and sealed with Teflon-coated butyl rubber septa aluminum crimp caps. When indicated, some of the medium components were omitted. At least three treatments were included for each experiment: (i) abiotic controls containing the anaerobic defined medium and nZVI, (ii) biotic controls containing the anaerobic defined medium and the corresponding bacterium, and (iii) biotic-abiotic treatments containing the anaerobic defined medium, nZVI, and the corresponding bacterium. Each microcosm was amended with either 1,2-DCA, CF or DCM (1,2-DCA from acetone stock solutions). In the biotic treatments, *Dehalobacterium* and *Dehalogenimonas* were inoculated by transferring 3 mL and 5 mL from cultures during the exponential degradation phase of DCM and 1,2-DCA, respectively. The microcosms containing nZVI were not gassed with H_2_ but with N_2_/CO_2_ (4:1, v/v, 0.2 bar overpressure). Biotic controls were cultivated under static conditions; biotic-abiotic treatments and abiotic controls were incubated under shaking conditions (150 rpm) with the aim of maintaining the nanoparticles in suspension. All treatments were maintained at 25 °C in the dark.

### Characterization of nZVI particles

The morphological features of nZVI and the interaction between cells and nZVI particles were analyzed with a Zeiss Merlin Field-Emission Scanning Electron Microscope (SEM). Samples were prepared as follows: a drop from the sample was placed in silicon chips, where it was then fixed with 2.5% glutaraldehyde in phosphate buffer 0.1 M for 2 h at 4 °C, post-fixed with 1% osmium tetroxide with 0.8% potassium ferrocyanide for 2 h, and dehydrated in increasing concentrations of ethanol (50, 70, 90, 96, and 100%). Finally, it was chemically dried with *hexamethyldisilazane*.

Samples were also analyzed by transmission electron microscopy (TEM) to measure the oxidation state of nZVI. A FEI Tecnai G2 F20 HR(S) was used. This TEM was equipped with a Gatan Image Filter (GIF) Quantum SE963 operated at 200 kV to obtain energy electron loss spectroscopy (EELS) analysis. This analysis defines an *L*_32_ ratio, which corresponds to a defined oxidation state of iron according to Tan et al. (2012). According to it, a value of 2.99 of this ratio corresponds to zero-valent iron, while oxidation states of + 2 and + 3 correspond to *L*_32_ values of 3.99 and 4.55, respectively.

### Analytical methods

Volatile halogenated compounds were analyzed by injecting 0.5 mL from the headspace samples into an Agilent 6890N gas chromatography (GC) fitted with an Agilent DB-624 column (30 m × 0.32 mm with 0.25 μm film thickness) and a flame ionization detector. Helium was used as the carrier gas (0.9 mL/min), and the injector and detector temperatures were set at 250 and 300 °C, respectively. To analyze CF, propene, *cis*-1,2-dichloroethene, vinyl chloride, chloroethane, methane, ethene, and 1,2-DCA, the initial oven temperature was 35 °C; it was held for 3 min and then ramped at 10 °C/min to 240 °C, which was held for 4 min (Trueba-Santiso et al. 2017). To analyze DCM, the initial temperature was 60 °C, and it was raised to 140 °C in 4 min. Then, the temperature was maintained at 140 °C for 1 min. Hydrogen was analyzed on 0.1-mL headspace samples using an Agilent 7820A GC equipped with a thermal conductivity detector. Columns used for separation were MolSieve 5A 60/80 SS (1.82 m × 2 mm, Agilent) and Porapak Q 60/80 UM (1.82 m × 2 mm, Agilent). The carrier gas was nitrogen at 138 kPa, and the oven temperature was held isothermal at 40 °C, the injector temperature at 200 °C, and the detector temperature at 250 °C. The pH was measured by taking 0.5 mL of liquid samples and using a pH probe HACH.

## Results and discussion

### Degradation of CF, DCM, and 1,2-DCA by nZVI

We first studied the abiotic dechlorination of CF, DCM, and 1,2-DCA by nZVI and the effect of omitting vitamins (V−) and sulfur-cysteine (S−) from the complete medium (CM) described in the “nZVI synthesis and bacterial cultures” section.

Based on previous reports, CF reacted with nZVI producing mainly DCM (Song and Carraway 2006). Therefore, we studied the optimal concentration of nZVI to transform 500 μM CF, which will be later used to determine the inhibitory effect of nZVI on the DCM-fermenting culture. The concentrations of nZVI tested were in the order of those typically applied in field studies: 0.05, 0.1, 0.5, and 1 g nZVI/L (Koenig et al. 2016; Wang et al. 2016; Dong et al. 2019).

**Fig. 1:**
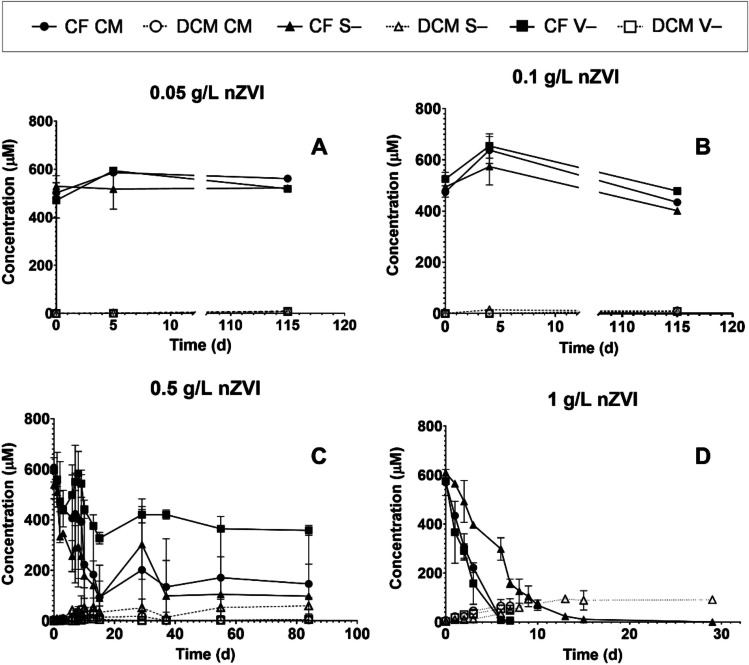
CF abiotic degradation and DCM production by nZVI at four different concentrations: **A** 0.05 g nZVI/L, **B** 0.1 g nZVI/L, **C** 0.5 g nZVI/L, and **D** 1 g nZVI/L. Three different culture media were used: complete medium (CM), vitamins lacking medium (V−), and sulfur-cysteine lacking medium (S−). Values plotted are means ± standard deviations for duplicate cultures

CF did not significantly react with nZVI when added at 0.05 and 0.1 g nZVI/L in any treatment (CM, V−, S−), and DCM was detected as a byproduct at very low concentrations (≤ 10 μM) after 115 days (Fig. 1A, B). In microcosms amended with 0.5 g nZVI/L (Fig. 1C), degradation of CF occurred in all treatments during the first 15 days, reaching a plateau since then. At this time point, the degradation of CF was ~ 80% in the CM and S− treatments and ~ 45% in the V− treatment. When the amended concentration was 1 g nZVI/L, full CF removal was achieved in 7 days in the CM and V- treatments, with a degradation rate of 80 ± 8 μM/d (Fig. 1D). When sulfur-cysteine lacked in the medium (S−), the degradation rate decreased at 20 ± 1 μM/d. The amount of DCM produced after the depletion of CF was 70 ± 30 μM DCM in CM and 50 ± 5 μM DCM in V−, which accounted for ~ 15 and 10% of the CF supplied, respectively. According to previous studies, solutes such as Cl^−^ or bicarbonate (present in our culture medium) can deactivate reaction sites in the nZVI surface (Tang et al. 2012; Han and Yan 2014). However, vitamins and sulfur-cysteine can act as electron shuttling compounds, hence enhancing electron transfer from nZVI to CF (Watanabe et al. 2009). This fact can explain the moderately slower reaction rate when sulfur-cysteine was missing in the culture medium. Because of this, the following microcosms were performed in complete culture media.

In addition to DCM, some other volatile compounds derived from CF degradation were detected at lower concentrations, including methane, ethene, propene, vinyl chloride, chloroethane, *cis*-1,2-dichloroethene, and 1,2-DCA (Table S1). These byproducts were also detected in previous studies employing nZVI to degrade chlorinated methanes (Song and Carraway 2006; Lee et al. 2015). Alternative byproducts described from CF degradation by nZVI that were not detected in this study include short-chain hydrocarbons (C1–C5) (Lee et al. 2015). The mass balance of the detected compounds accounted for ~ 30% of the CF supplied. In addition to the unidentified compounds, a closed mass balance is difficult because some of the generated products can be adsorbed on the surface of the nanoparticles (Lee et al. 2015).

Based on the above results, the abiotic degradation of DCM (2000 μM) and 1,2-DCA (500 μM) was assessed using 1 g nZVI/L. Consistent with previous reports (Gillham and O’Hannesin 1994; Song and Carraway 2005; Koenig et al. 2016; Lee et al. 2015), there was no significant reactivity of nZVI toward both 1,2-DCA and DCM (Fig. S1). Thus, these pollutants remained in the medium and needed alternative remediation treatments, which opens the window to their biodegradation using anaerobic dechlorinating bacteria.

### Effect of nZVI on Dehalobacterium activity

#### Impact of nZVI

As stated before, biodegradation of DCM under anoxic conditions has been reported solely in three bacterial genera. Since DCM is the by-product from CF degradation by nZVI but it is not degraded further by this treatment, we studied whether nZVI has a toxic effect on microbial transformation of DCM (2000 μM) by a *Dehalobacterium*-containing culture. For this purpose, we used the two nZVI concentrations, which were found to fully remove CF in previous tests: 0.5 and 1 g nZVI/L (Fig. 1).

**Fig. 2:**
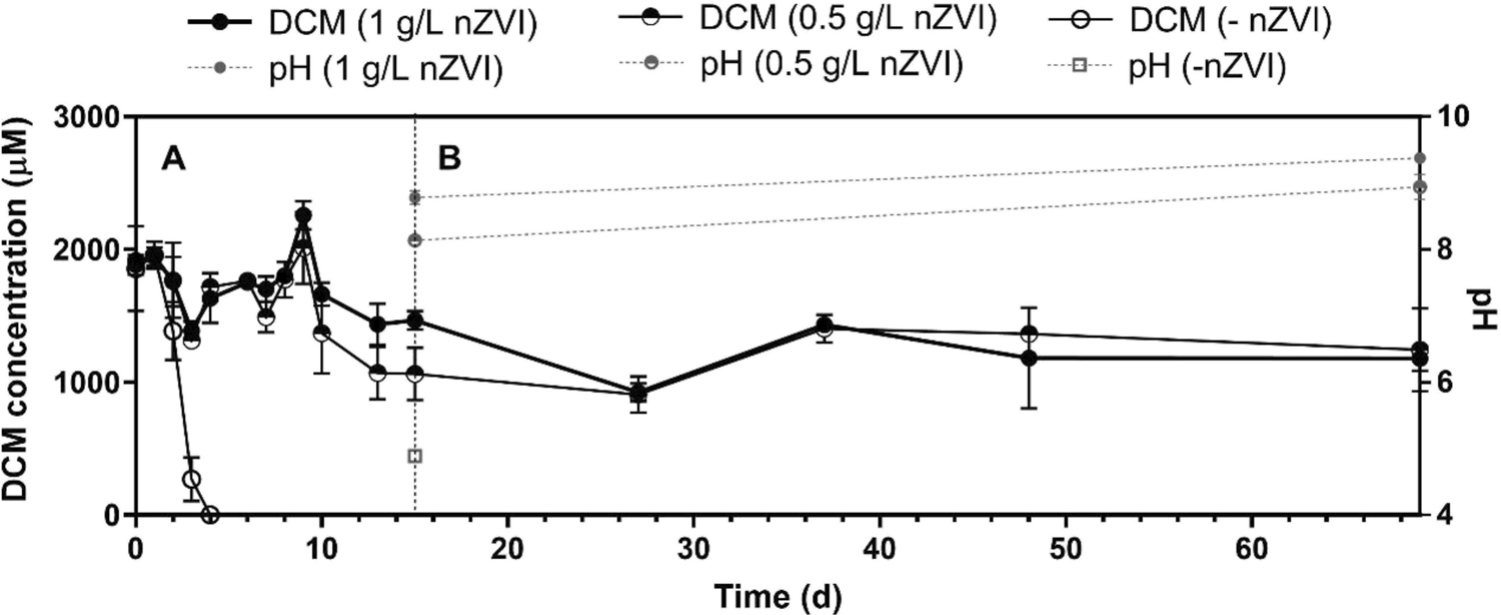
Time course of DCM degradation by a *Dehalobacterium*-containing consortium in a complete anaerobic culture medium with 0.5 and 1 g nZVI/L. This test presents two phases: **A** cultures under shaken conditions (150 rpm) and **B** cultures with nZVI precipitated under static conditions. Biotic controls (-nZVI) consisted of *Dehalobacterium*-containing cultures amended with DCM without nZVI. Values plotted are means ± standard deviations for triplicate cultures

DCM fermentation by *Dehalobacterium* was not observed with either 0.5 or 1 g nZVI/L, while biotic microcosms fully fermented DCM in 4 days (Fig. 2A). It indicates that nZVI exerts an inhibitory effect on *Dehalobacterium.* Then, the next step was exploring the factor that provokes the inhibitory effect.

#### Adsorption of nZVI on bacterial cells

**Fig. 3:**
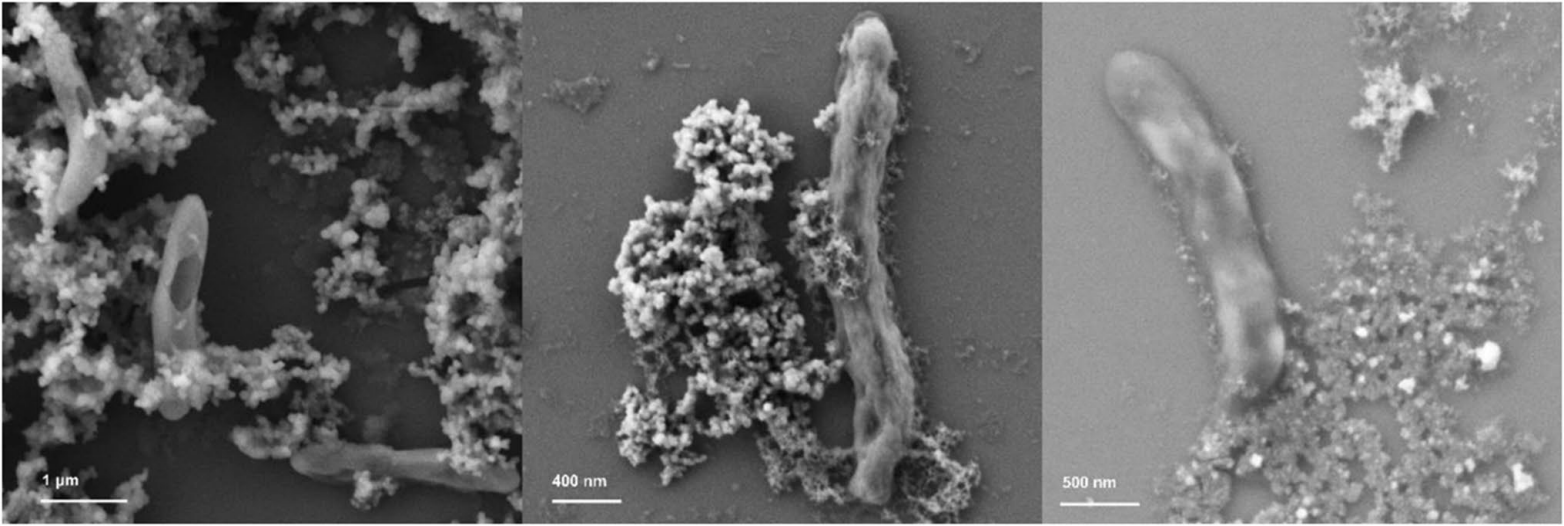
SEM micrographs from *Dehalobacterium*-containing cultures with 1 g nZVI/L

**Fig. 4:**
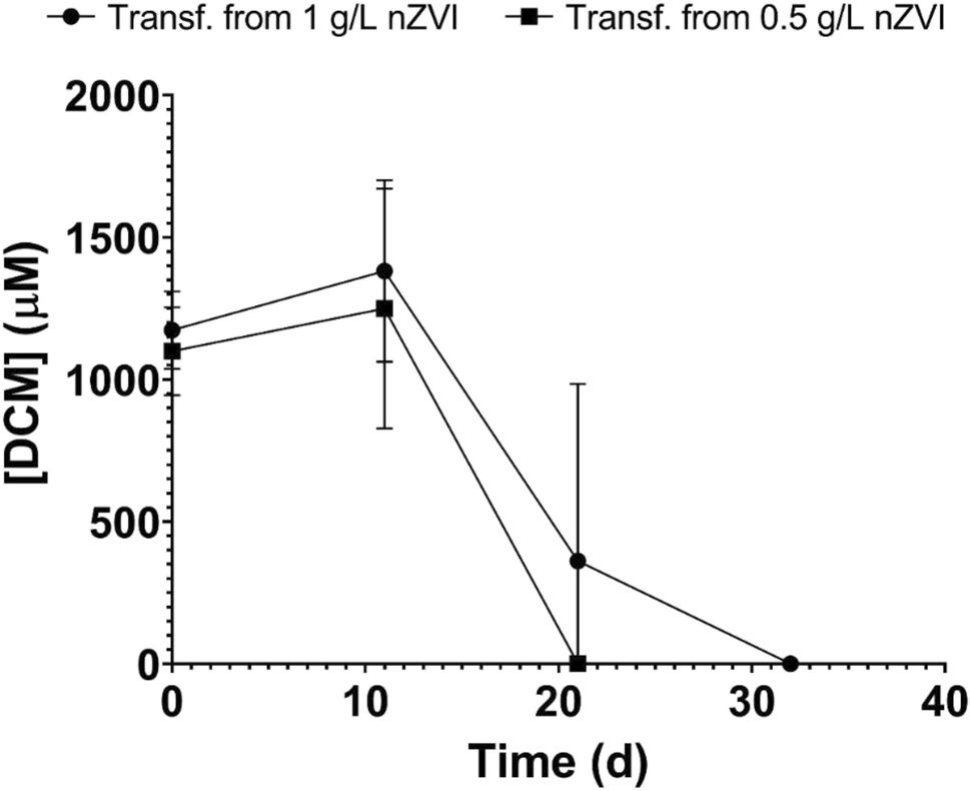
Time course of DCM degradation by a *Dehalobacterium*-containing consortium after being transferred to fresh complete medium from microcosms containing nZVI. Values plotted are means ± standard deviations for triplicate cultures

It has been reported that nZVI can provoke physical disruption of cell membranes. Although this mechanism is not fully understood, it has been hypothesized that it is primarily due to the chemical interaction between cells and the reactive surface of nZVI (e.g., iron is a strong reductant), which could favor the decomposition of functional groups in proteins and lipopolysaccharides from cell membranes (Lee et al. 2008; Li et al. 2010). SEM analysis revealed a deformed cell structure with adsorption of nZVI on the cell surface with 1 g nZVI/L (Fig. 3). To study whether nZVI adsorbed on cell membranes caused the inhibition, on the fifteenth day, we stopped shaking the cultures and precipitated nZVI at the bottom of the serum bottles using a magnet, while microorganisms remained in suspension, thus avoiding contact between nZVI and *Dehalobacterium* (Fig. 2B). In the following 55 days, no degradation of DCM was observed in the bottles with 0.5 and 1 g nZVI/L (Fig. 2B). Also, on the fifteenth day, 3 mL of both cultures containing 0.5 and 1 g nZVI/L were transferred to fresh complete culture media without nZVI and amended with DCM (1000 μM). DCM was completely degraded after 20–30 days, with a lag phase of 10 days (Fig. 4). These results suggested that inhibition of DCM fermentation by nZVI was reversible, and that nZVI adsorption to cells was not the main cause of the inhibition.

#### pH

**Fig. 5:**
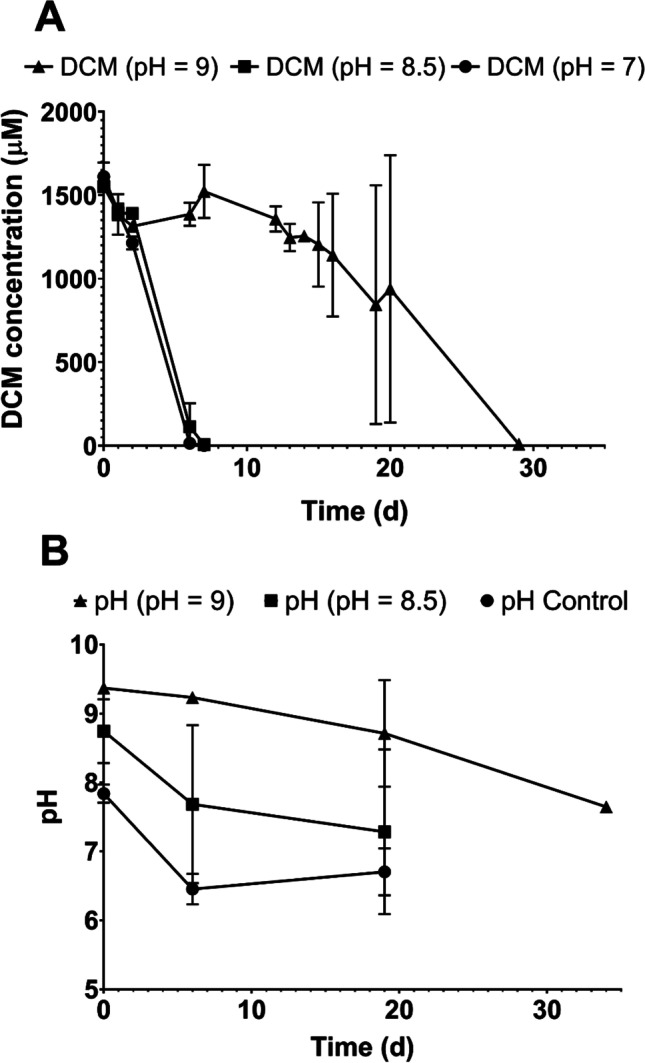
**A** Time course of DCM degradation by a *Dehalobacterium*-containing consortium in complete anaerobic culture media at different pH values (7, 8.5, and 9). **B** Changes in pH during DCM degradation. Values plotted are means ± standard deviations for triplicate cultures

The increase in pH due to corrosion of nZVI can negatively impact *Dehalobacterium* activity. pH on day 65 increased in cultures amended with nZVI (8.8 ± 0.1 with 1 g nZVI/L and 8.1 ± 0.1 with 0.5 g nZVI/L) compared with cultures without nZVI (4.9 ± 0.1) (Fig. 2). To determine whether DCM fermentation was inhibited in a basic medium, we established new *Dehalobacterium* cultures at pH 7 (control), 8.5, and 9 without nZVI. Degradation of DCM in cultures at pH 7 and 8.5 occurred at similar degradation rates after a lag phase of 6 days, but cultures at pH 9 exhibited an extended lag phase of 18 days before DCM was fully consumed (Fig. 5). Interestingly, cultures at pH 9 (9.4 ± 0.1) did not start degrading DCM until pH reached a value of 8.7 ± 0.8 due to the slow degradation of DCM that acidified the medium because the production of acetate and H^+^ (Trueba-Santiso et al. 2020).

**Fig. 6:**
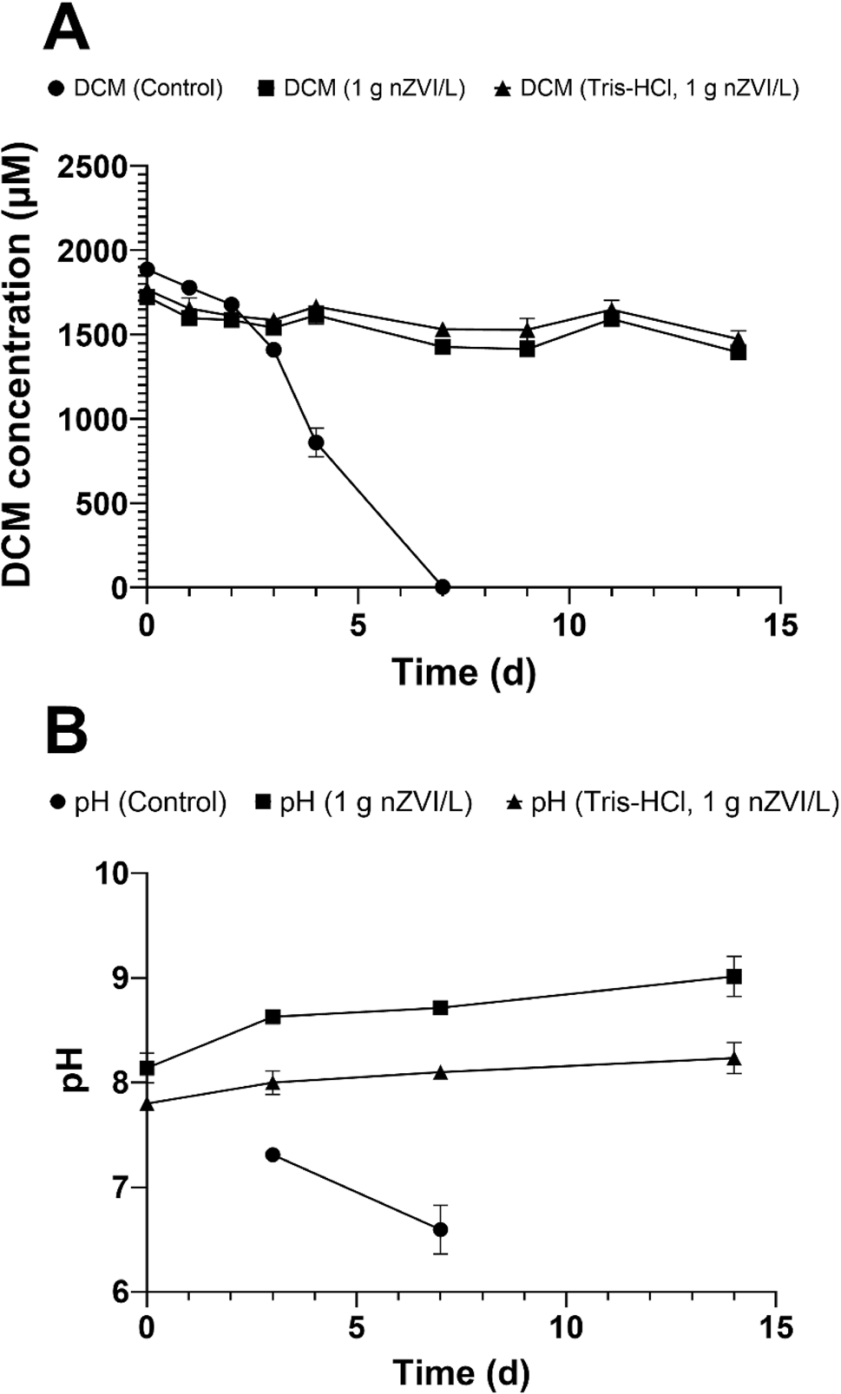
**A** Time course of DCM degradation by a *Dehalobacterium*-containing consortium in complete anaerobic culture media with 1 g nZVI/L and buffered and non-buffered with 25 mM Tris-HCl. **B** Changes jn pH during DCM degradation. Values plotted are means ± standard deviations for duplicate cultures. Controls consisted of *Dehalobacterium*-containing cultures amended with DCM without nZVI

To avoid the negative impact of high pH values on *Dehalobacterium* activity, we buffered the *Dehalobacterium*-containing cultures with 25 mM Tris-HCl (pH = 7.5) and amended 1 g nZVI/L, but degradation of DCM was not observed for 12 days although pH was 8.2 ± 0.3 (Fig. 6). Cultures that were not buffered with Tris-HCl reached pH = 9.4 ± 0.4, whereas controls that did not contain nZVI degraded DCM in 7 days (Fig. 6). These results confirm that although degradation of DCM is delayed at pH values up to 9, other factors contributed to the cytotoxicity of *Dehalobacterium* when exposed to nZVI.

#### Effect of hydrogen and soluble compounds

Elevated H_2_ partial pressures have been found to inhibit the fermentation of DCM (Chen et al. 2017b; Lee et al. 2012; Trueba-Santiso et al. 2020). The reason for this inhibitory effect is still unclear. According to Chen et al. (2017b), this fact could be due to the hindrance of formate oxidation at such hydrogen concentrations, leading to the accumulation of formate into the cells of “*Candidatus* Dichloromethanomonas elyuquensis.” Hydrogen reached concentrations of 1600 ± 100 μM and 15000 ± 3000 μM when nZVI was amended at 0.5 and 1 g nZVI/L on day 15 in the experiment depicted in Fig. 2, and were dramatically higher compared with 23 ± 3 μM H_2_ obtained in the biotic microcosms (without nZVI). It provides evidence that H_2_ accumulated because of water hydrolysis by nZVI even though hydrogenotrophs were present in the bacterial consortium (Trueba-Santiso et al. 2020).

**Fig. 7:**
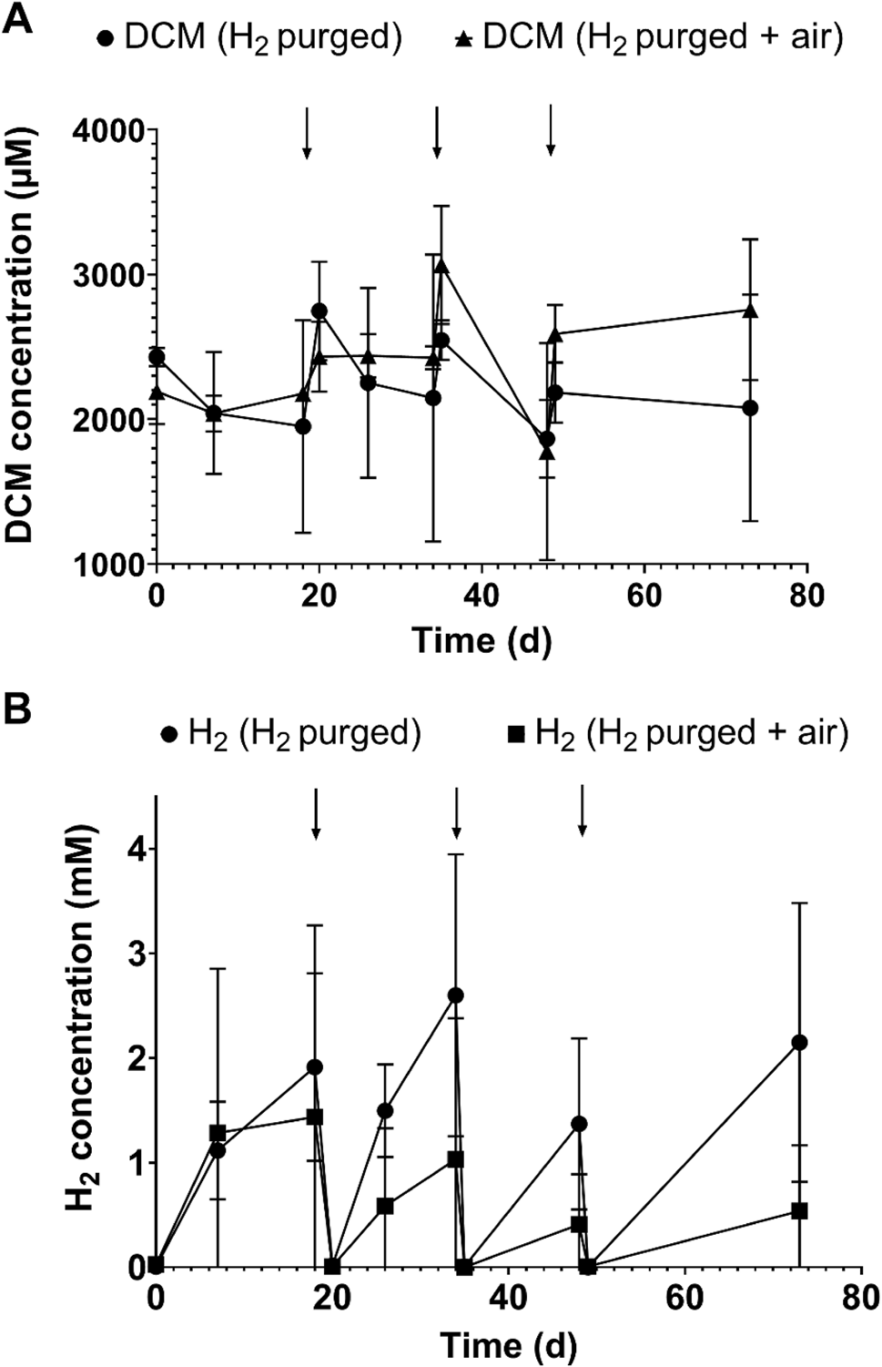
**A** Effect of purging hydrogen with nitrogen (H_2_ purged) and nitrogen plus air (H_2_ purged + air) on DCM degradation and **B** H_2_ generation in *Dehalobacterium*-containing cultures buffered with Tris-HCl and amended with 1 g nZVI/L. Arrows indicate when H_2_ was purged. Values plotted are means ± standard deviations for duplicate cultures

To study whether H_2_ exerted an inhibitory effect on *Dehalobacterium* activity in the cultures buffered with Tris-HCl and amended with 1 g nZVI/L, we periodically removed H_2_ after day 12 by purging with nitrogen (Fig. 7). DCM was then reamended (2000 μM). In addition, a second treatment in parallel cultures consisted of introducing air (1 mL) after purging with nitrogen to decrease the reductant power of nZVI and reduce the H_2_ production. Although the production of H_2_ decreased in cultures amended with air, H_2_ was rapidly generated after each purge (Fig. 7). After 70 days, H_2_ produced exceeded the concentration of 3000 ppmv (126 μM) found inhibitory for DCM fermentation in *Dehalobacterium* (Chen et al. 2017b).

Due to the continued production of H_2_ by nZVI, it was difficult to establish whether the high partial pressure of H_2_ was the only factor responsible for the inhibition of DCM fermentation. Therefore, we assessed whether hydrogen or a soluble inhibitory compound from nZVI were inhibitory factors in the *Dehalobacterium* activity.

**Fig. 8:**
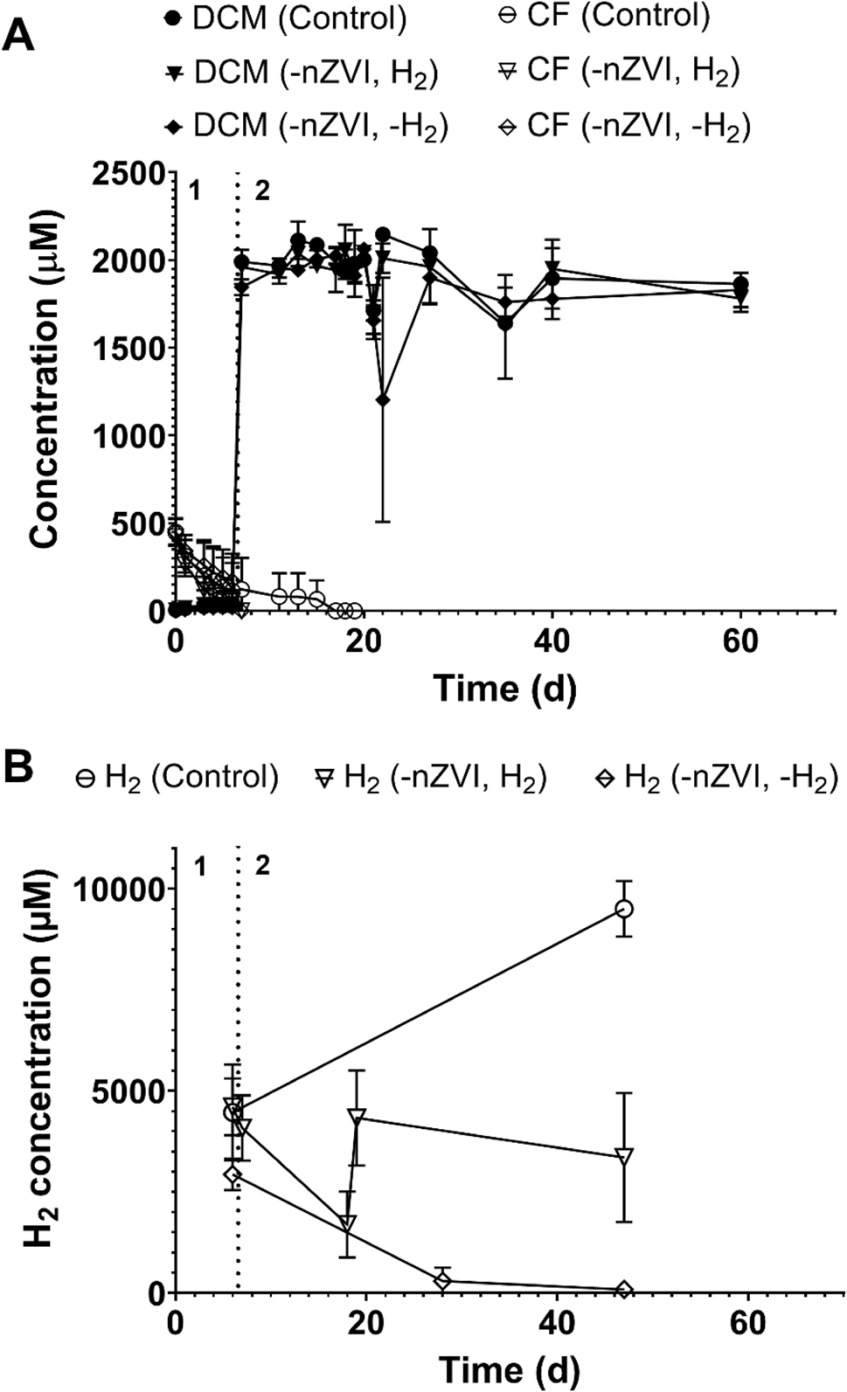
**A** Time course of abiotic-biotic degradation of CF by 1 g nZVI/L in *Dehalobacterium*-containing cultures (1). Time-course degradation of DCM in *Dehalobacterium*-containing cultures after the removal of nZVI with (-nZVI, H_2_) and without (–nZVI, –H_2_) amended H_2_ (2). **B** Hydrogen produced by nZVI. Values plotted are means ± standard deviations for triplicate cultures

We prepared nine abiotic-biotic microcosms containing *Dehalobacterium* and 1 g nZVI/L in a complete medium buffered with 25 mM Tris-HCl and 500 μM CF. When CF was depleted by nZVI on day 7 (Fig. 8), three different treatments were established in triplicate: i) particles of nZVI were removed from microcosms using a magnet, but cultures were gassed with H_2_ at the concentration reached in the microcosms before ZVI removal (–nZVI, H_2_), ii) particles of nZVI were removed from microcosms with no addition of H_2_ (–nZVI, –H_2_), and iii) particles of nZVI were not removed (control). All treatments were amended with 2000 μM DCM after CF depletion.

On day 7, H_2_ concentration was 4 ± 1 mM (Fig. 8), and pH was 7.8 ± 0.1 in all treatments. No degradation of DCM was observed in the following 53 days for any of the three treatments. Hydrogen was steadily consumed in cultures that did not contain nZVI to levels below the inhibition concentration (Fig. 8), but DCM was not degraded, providing additional supporting evidence that dissolved compounds derived from nZVI could contribute to the inhibition of *Dehalobacterium*. It has been described that soluble substances released or produced by nZVI particles that can cause inhibition to bacterial cells include radical species (i.e., hydroxyl radicals) (Anang et al. 2021; Xie et al. 2017), soluble iron (Fajardo et al. 2013; Koenig et al. 2016; Lee et al. 2008; Rangan et al. 2020) and soluble metals (Xie et al. 2017). Soluble iron can enter into the cells and generate ROS species by Fenton reactions, which could lead to damage by oxidative stress (Fajardo et al. 2013; Lee et al. 2008; Saccà et al. 2014).

On day 17, 3 mL of each microcosm was transferred to fresh culture media plus DCM (2000 μM), and complete degradation of DCM was observed after a lag phase of 14 ± 3 days in microcosms derived from treatment “–nZVI, –H_2_” (Fig. S2), indicating that inhibition was reversible.

Further research is needed to identify the inhibitory compound or compounds released or catalyzed by nZVI to overcome the inhibition of DCM degradation in *Dehalobacterium*. Some strategies to attenuate such inhibitory effect include the surface modification of nanoparticles with surface stabilizers such as surfactants or polyelectrolytes, which slow down nZVI reaction rates, the production of H_2_, the release of ROS and iron ions, and avoid contact between nZVI particles and cells (Xie et al. 2017; Wang et al. 2016). For instance, Kocur et al. (2016) detected an increase in the *Dehalogenimonas* population after the injection of 1 g nZVI/L stabilized with carboxymethyl cellulose in a contaminated aquifer. A similar phenomenon was observed for the ORB *Dehalococcoides mccartyi* (Xiu et al. 2010; Kocur et al. 2015).

#### nZVI characterization

**Fig. 9:**
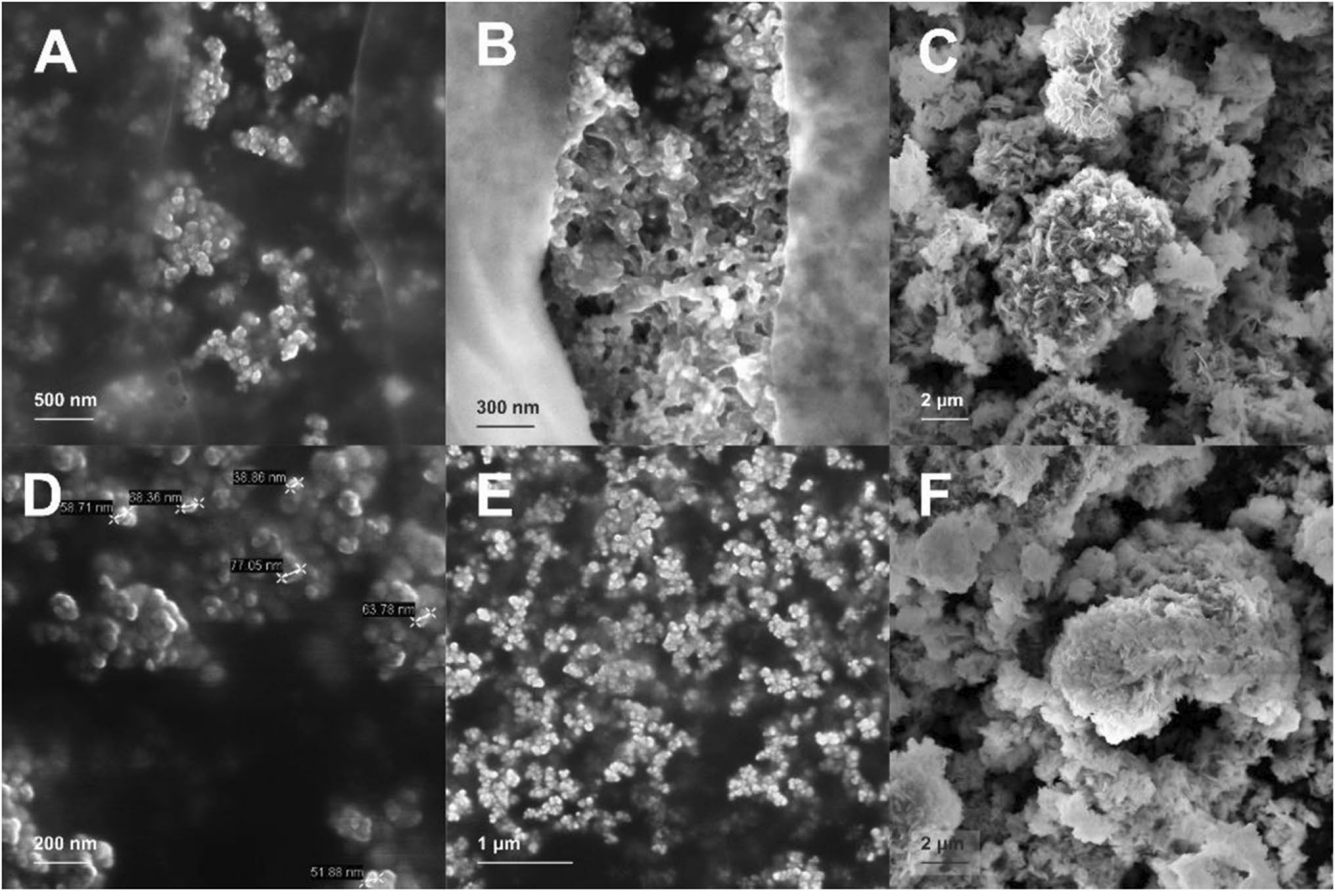
SEM micrographs of nZVI from abiotic treatments (CF + nZVI) (**A** to **C**) and abiotic-biotic treatments (DCM + *Dehalobacterium*+ nZVI) (**D** to **F**) at different stages: initial time (**A** and **D**), half time (**B** and **E**), and final time (**C** and **F**)

The morphological characterization of nZVI from two different treatments (CF + nZVI, shown in Fig. 9A–C and DCM + *Dehalobacterium*-containing culture + nZVI, shown in Fig. 9D–F) was performed with SEM. Samples were taken in three different stages: beginning (*t* = 0, Fig. 9A and D), mid-term (Fig. 9B and E), and final time (when the experiments were finished, Fig. 9C and F). Mid-term was the third day for microcosms containing CF + nZVI and the eighth day for the microcosms containing *Dehalobacterium*. At time zero, nZVI were spherical or near spherical in shape (50–100 nm) (Fig. 9A and D) and eventually formed aggregates in the mid-term stage (Fig. 9B and E). Aggregation of nZVI and the subsequent formation of a particle network (gelation) have been pointed to limit transport of nZVI in water-saturated porous media (Phenrat et al. 2007). At the end of abiotic and biotic-abiotic experiments, nZVI showed a rough surface topography, which is consistent with the formation of iron oxides and hydroxides on the surface of nZVI (Fig. 9C and F).

The oxidation state of the nZVI particles used in some of the treatments carried out in this study was determined by EELS analysis. Each treatment showed an oxidation state ≥ + 3 at the end of the experiment according to the *L*_32_ ratio values (Table 1), indicating oxidation of nZVI through the experiments is in accordance with micrographs depicted in Fig. 9.

**Table 1:** *L*_32_ index of samples from experimental microcosms containing nZVI. Sample 1 derived from the experiment depicted in Fig. 1C on day 7; sample 2 derived from the experiment depicted in Fig. 8 on day 7; sample 3 derived from the experiment depicted in Fig. 2 on day 15; sample 4 derived from the experiment depicted in Fig. 6 on day 12

	***L***_***32***_ ***index values***
*1) CF + 0.5 g nZVI/L*	*4.9*
*2) CF + Dehalobacterium + 1 g nZVI/L+ Tris-HCl*	*5.77*
*3) DCM + Dehalobacterium + 1 g nZVI/L*	*5.34*
*4) DCM + Dehalobacterium + 1 g nZVI/L + Tris-HCl*	*4.56*

### Effect of nZVI on Dehalogenimonas activity

**Fig. 10:**
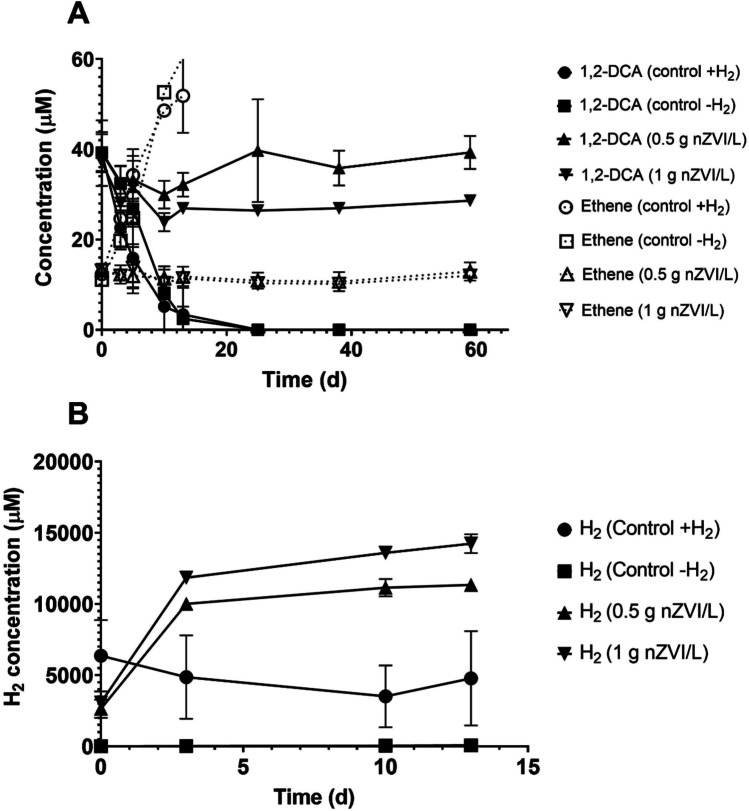
**A** Time course of 1,2-DCA degradation by 0.5 and 1 g nZVI/L in *Dehalogenimonas*-containing cultures. Ethene production is also shown. **B** H_2_ concentrations in microcosms with *Dehalogenimonas*-containing cultures. Values plotted are means ± standard deviations for triplicate cultures

As shown in Fig. S1, 1,2-DCA was not removed by 1 g nZVI/L. Because of this, we aimed to test the capability of a *Dehalogenimonas*-containing culture to dechlorinate 1,2-DCA in the presence of nZVI using a HEPES buffered medium. Four different treatments were tested: i) biotic microcosms (control + H_2_), ii) biotic microcosms without H_2_ (control -H_2_), iii) abiotic-biotic microcosms containing 0.5 g nZVI/L, and iv) abiotic-biotic microcosms containing 1 g nZVI/L. Both control treatments removed 1,2-DCA at the same rate with the concomitant production of ethene, while those microcosms containing nZVI did not show 1,2-DCA depletion despite the circumneutral pH and the high production of H_2_ that could be used as electron donor for *Dehalogenimonas* (Fig. 10). Therefore, we transferred 5 mL of the microcosms to fresh culture media with 1,2-DCA (50 μM) to test whether the inhibitory effect was reversible, and full removal of 1,2-DCA was observed in 50 days after a lag phase of ~ 20 days (Fig. S3). This is in line with the tests performed by Koenig et al. (2016), indicating that concentrations of nZVI supplied above 0.5 g/L inhibited the dechlorination activity of groundwater containing *Dehalogenimonas* likely due to ROS species).

Despite the inhibition of *Dehalogenimonas* activity by nZVI, our results are not necessarily transferable to field studies. The high complexity of the soil and groundwater composition could avoid the direct contact between nanoparticles and cells while providing optimum conditions for ORB growth (low redox potential, H_2_ production).

## Conclusions

Our laboratory tests demonstrated that dechlorinating activity of *Dehalobacterium* and *Dehalogenimonas* was inhibited at 1 g nZVI/L, which was the optimal concentration of nZVI found to fully remove CF (500 μM). Further experiments discarded that changes in both pH and partial pressure of H_2_ provoked by nZVI exerted inhibition on *Dehalobacterium*. The fact that both *Dehalogenimonas* and *Dehalobacterium* recovered the dechlorinating capability after being transferred to nZVI-free medium pointed to soluble compounds released by particles as potential inhibitors. These findings have implication for remediation strategies aiming to combine both abiotic and biotic treatments to decontaminate aquifers with mixtures of chlorinated compounds. In particular, our study suggests that factors such as the buffering capacity of groundwaters and dilution processes can play crucial roles in the viability of this combined strategy in bioremediation treatments.

## Supplementary information

ESM 1 (DOCX 236 kb)
