# Supplementary material for: Combining nanoscale zero-valent iron and anaerobic dechlorinating bacteria to degrade chlorinated methanes and 1,2-dichloroethane
Source: Environ Sci Pollut Res Int. 2023 Jan 27;30(15):45231–43. doi: 10.1007/s11356-023-25376-z (PMC10076415; doi:10.1007/s11356-023-25376-z)
Supplement: Supplementary file 1 — (DOCX 236 kb) [file 11356_2023_25376_MOESM1_ESM.docx]

# **Supporting information**

# Combining nanoscale zero-valent iron and anaerobic dechlorinating bacteria to degrade chlorinated methanes and 1,2-dichloroethane

Dani Salom^1^, David Fernández-Verdejo^1^, Javier Moral-Vico^1^, Xavier Font^1^, Ernest Marco-Urrea^1,*^

## ^1^Departament d’Enginyeria Química, Biològica i Ambiental, Universitat Autònoma de Barcelona (UAB), 08193 Bellaterra, Barcelona, Spain.

* Corresponding author: Ernest Marco-Urrea. Departament d'Enginyeria Química, Biològica i Ambiental, Universitat Autònoma de Barcelona (UAB), 08193 Bellaterra, Barcelona, Spain. Phone: +34 935812694; Email: ernest.marco@uab.es.

**FIGURES**

**
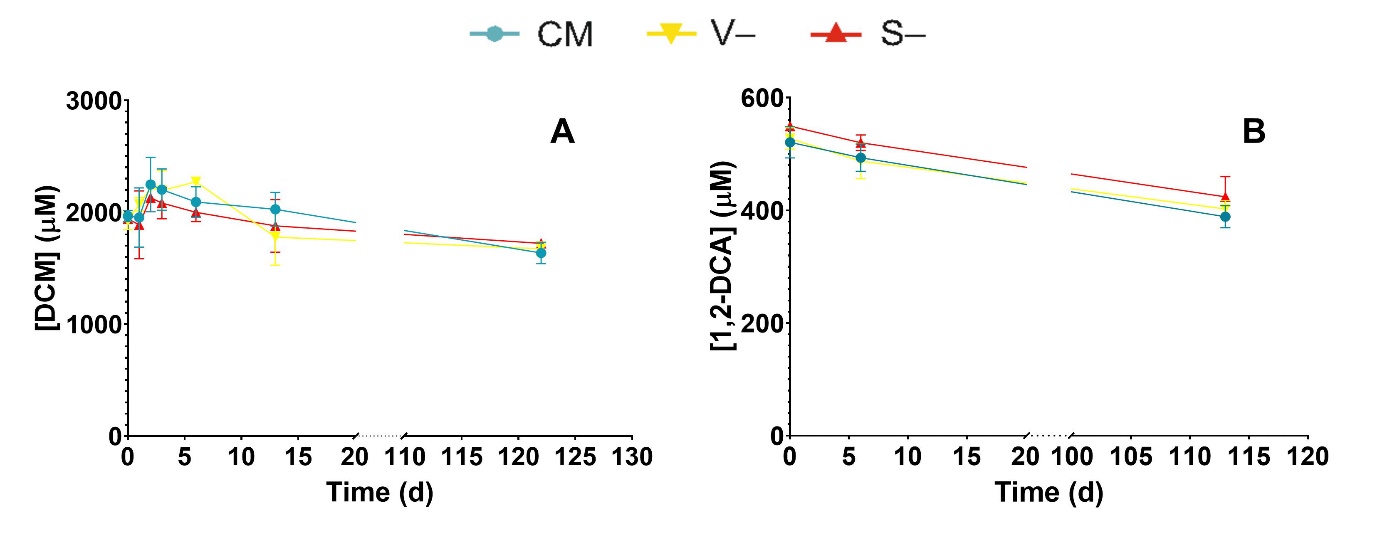
**

**Fig. S1.** Abiotic DCM and 1,2-DCA dechlorination using 1 g nZVI/L in three different anaerobic culture media: complete culture medium (CM), vitamins lacking medium (V-) and sulfur-cysteine lacking medium (S-). Values plotted are means ± standard deviations for triplicate cultures.


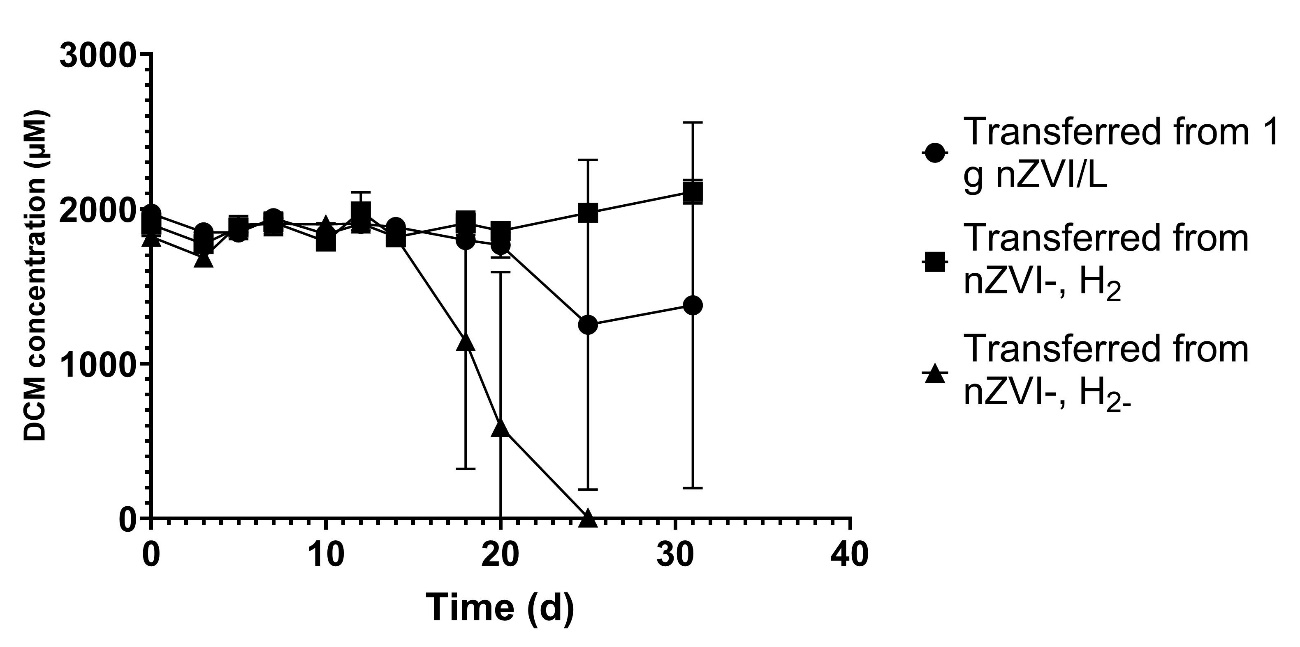


**Fig. S2.** DCM fermentation carried out by *Dehalobacterium* consortia transferred to fresh medium from microcosms with three different treatments after 7 days exposed to 1 g nZVI/L: i) particles of nZVI were removed from microcosms using a magnet, but cultures were gassed with H_2_ at the concentration reached in the microcosms before nZVI removal (nZVI-, H_2_), ii) particles of nZVI were removed from microcosms with no addition of H_2_ (nZVI-, H_2_-), and iii) particles of nZVI were not removed (control). Values plotted are means ± standard deviations for triplicate cultures. All treatments were amended with 2000 µM DCM.

**
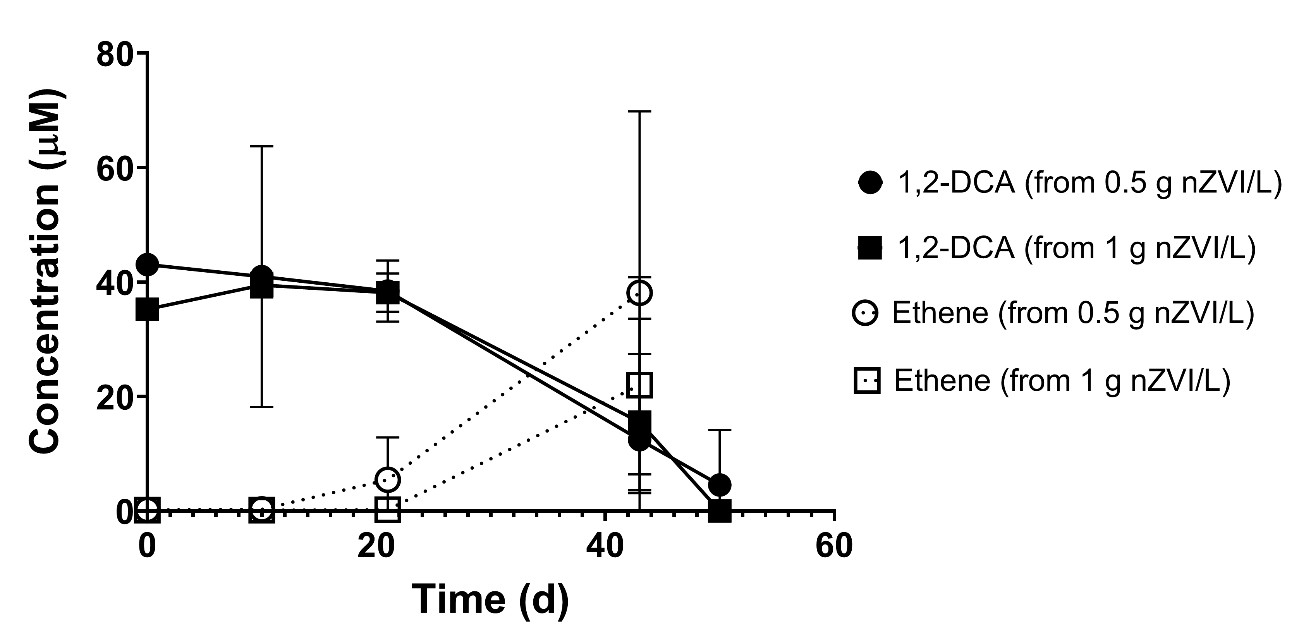
**

**Fig. S3.** 1,2-DCA removal and ethene production carried out by *Dehalogenimonas* consortia transferred from microcosms containing 0.5 and 1 g nZVI/L. Values plotted are means ± standard deviations for triplicate cultures.

**TABLES**

**Table S1.** By-products detected in abiotic treatments amended with CF and nZVI. Values are means ± standard deviations for duplicate cultures.

| **By-product** | **Concentration (µM) (0.5 g nZVI/L)** | **Concentration (µM) (1 g nZVI/L)** |
| --- | --- | --- |
| Dichloromethane | 50 ± 50 | 70 ± 30 |
| Methane |  | 60 ± 10 |
| Ethene |  | 18 ± 6 |
| Propene | 2 ± 3 | - |
| cis-1,2-dichloroethene | 3 ± 1 | 2 ± 1 |
| Vinyl chloride | 7 ± 9 | 16 ± 2 |
| Chloroethane | 1 ± 1 | 2 ± 1 |
| 1,2-dichloroethane | - | 1 ± 1 |
